# Intraluminal Vesicles as Transfection Intermediaries

**DOI:** 10.3390/pharmaceutics17121584

**Published:** 2025-12-09

**Authors:** Nourhan A. M. Mahmoud, Hadeer K. S. Abdelrahman, Benedita K. L. Feron, Andra Pintilie, Marc Fivaz, Joanna J. Miest-Bray, Timothy Gomez, Natalie Youens, Vineeta Tripathi, Simon C. W. Richardson

**Affiliations:** 1 The Exogenix Laboratory, School of Science, University of Greenwich, Central Avenue, Chatham Maritime, Kent ME4 4TB, UK; nalaaeldin@msa.edu.eg (N.A.M.M.); hkhyal@msa.edu.eg (H.K.S.A.); benedita.feron@umu.se (B.K.L.F.); an3202p@greenwich.ac.uk (A.P.); timothy.gomez@greenwich.ac.uk (T.G.); n.c.youens@greenwich.ac.uk (N.Y.); 2 Department of Biochemistry, Faculty of Pharmacy, October University for Modern Sciences and Arts (MSA), Giza 12451, Egypt; 3 Department of Chemistry, Umeå University, 901 87 Umeå, Sweden; 4 Remynd, Bio-Incubator, Gaston Geenslaan 1, 3001 Leuven, Belgium; marc.fivaz@remynd.com; 5 The School of Sciences, Canterbury Christ Church University, Anselm Building, North Holmes Road, Canterbury, Kent CT1 1QU, UK; joanna.miestbray@canterbury.ac.uk; 6 Vitarka Therapeutics Ltd., Innovation House, Discovery Park Sandwich, Kent CT13 9FF, UK; vineeta.tripathi@vitarka.co.uk

**Keywords:** anthrax toxin, drug delivery, siRNA, endocytosis, intraluminal vesicle

## Abstract

**Background:** To address hepatotropic body distribution and toxicity, transfection systems based on protein architecture have been proposed. Attenuated anthrax toxin (aATx) has provided the backbone for a first in class transfection system that, in the wild, uses intraluminal vesicles (ILVs) as an intermediary compartment during the translocation of large molecules into the cytosol. Small interfering (si)RNA molecules non-covalently attached to a carrier (LFn-PKR) would not be predicted to be an aATx translocase (protective antigen (PA)) substrate. Previously, siRNA has been shown to be delivered to the cytosol using this system. **Methods:** Here, the localisation of ^32^P-labelled siRNA delivered using aATx was quantified directly and related to siRNA activity. In addition, inhibition of ILV formation by hypertonic sucrose or wheatgerm agglutinin (WGA) was shown to inhibit the aATx-mediated cytosolic translocation of siRNA. **Results:** MCF-7 cells were used to establish siRNA intracellular distribution in relation to pharmacological activity by targeting STAT3 gene expression. After Lipofectamine-mediated transfection using 100 nM ^32^P-labelled siRNA, 45 ± 3.2% (±SD; *n* = 3) of the cell associated siRNA was found in the cytosol. After the transfection of 100 nM ^32^P-labelled siRNA using aATx, 77 ± 2.5% (±SD; *n* = 3) of the cell associated siRNA was found in the cytosol and resulted in a reduction in STAT3 expression of 64.04 ± 14.17% (±SD; *n* = 3) relative to an untreated control by Western analysis. Further, 25 μg/mL of WGA inhibited 75.23 ± 0.06% (±SD; *n* = 3) of the knockdown attributed to a non-WGA-treated control. Relative to the control, treatment with 200 mM sucrose resulted in a reduction of 74.58 ± 7.76% (±SD; *n* = 3) of target gene knockdown. **Conclusions:** These data indicated that the insertion of the PA pore into endosomal membrane did not weaken the endosomal limiting membrane, leading to vesicular bursting during transfection and ILVs played critical role in translocase activity.

## 1. Introduction

The widespread use of transfection systems mediating RNA delivery for vaccination against SARS-CoV-2 underscore the utility of RNA-based medicines [1]. However, the success of these systems has also highlighted the limitations associated with RNA drug delivery technologies. Whilst drugs mediating the delivery of synthetic mRNA to striated muscle and small interfering (si)RNA to the liver have met with regulatory approval, delivery to other tissues remains challenging [2,3]. As these limitations are driven by physiochemistry, new systems have been explored. One such system utilises architecture that has evolved to mediate cytosolic drug delivery and is based on an attenuated anthrax toxin (aATx) [4,5,6,7,8]. Questions remain about the mechanisms that this system uses to deliver cargo across the cell membrane. Wild-type ATx has been reported to utilise intraluminal vesicles (ILVs) formed within endosomes to facilitate the destruction of the phosphorylated (abluminal) portion of activated mitogenic receptors [9]. ILVs also serve as an intermediary compartment prior to the delivery of approximately 80% of internalised lethal factor (LF), internalised as anthrax lethal toxin to the cytosol [10]. Translocation into the lumen of the ILV within the endosome requires protective antigen (PA) protein, which, after hydrolytic activation and oligomerisation, acts as a cation-selective translocase that requires the “unfolding” of translocase substrates [11]. Substrate unfolding, also known as a molten globular transition, has been reported to be critical for the translocase substrate to move across the phenylalanine clamp at the base of the PA vestibule, which has an internal diameter of ~6 Å [11]. We previously reported the use of an aATx system to deliver specific proteins, antisense oligonucleotides (ASOs), and siRNA to the cytosol of mammalian cells, with high efficiency and low toxicity [4]. For siRNA and ASO delivery, substrate unfolding would dislocate anionic siRNA or ASOs from the LF-derived shuttle protein. Consequently, our previous work indicated that under certain conditions, it was possible that the PA pore may radially expand, as its lattice-like structure might suggest, instead of the cargo unfolding completely. Herein, we characterise this system further and identify ILVs as transfection intermediaries. The shuttle proteins used herein consisted of the non-toxic N-terminal region (amino acids 25-255) of ATx Lethal Factor (LF) (referred to as LFn), fused-in-frame with sequence coding for the RNA-binding domain of protein kinase R (LFn-PKR) [4]. As the diameter of the phenylalanine clamp would, if static, prohibit the passage of anionic siRNA and the unfolding of the LFn-PKR would dislocate it from non-covalently attached cargo, an investigation into the trafficking mechanisms responsible for transfection using the aATx system [4] was undertaken. To this end, it was asked if siRNA could be detected in the cytosol (directly) after subcellular fractionation, rather than relying on an indirect measure of siRNA translocation such as (i) pharmacological activity [4,8] or (ii) the presence of LFn-PKR or a fluorescently labelled LFn derivative [4] or (iii) fluorescently labelled siRNA [8]. To quantitate the uptake and intracellular distribution of siRNA, the covalent labelling of siRNA was undertaken using ^32^P. The use of radionucleotides was necessary, as the sensitivity of the fluorescence assays we had previously explored was not sufficient for a quantitative rather than qualitative estimation of siRNA subcellular distribution [8].

To investigate the role of ILVs during aATx-mediated transfection, two inhibitors of ILV formation and one of endocytic vesicle fusion were assayed for an effect upon PA translocase activity. Recombinant LFn fused-in-frame with diphtheria toxin a chain (LFn-DTA), previously shown to be a PA pore substrate [12], served as a positive control for PA-mediated translocation, and LFn-PKR non-covalently attached to siRNA [4] was used to evaluate the translocation of siRNA complexes. The inhibitors evaluated were the lectin wheatgerm agglutinin (WGA), which binds to *N*-acetylglucosamine and sialic acid residues on the plasma membrane [13], crosslinking the internalised cell membrane and leading to an expansion in the diameter of endocytic vesicles at higher concentrations [14,15]; wortmannin, a fungal metabolite and potent inhibitor of phosphoinisitol (PI)3 kinase required for early endocytic vesicle fusion [16]; and hypertonic sucrose, previously shown to increase vesicle intraosmotic pressure [17,18]. We reasoned that WGA-mediated membrane cross-linking would limit the ability of the endosomal sorting complex required for transport (ESCRT) to drive ILV biogenesis and invagination into the endosomal lumen [19]. Further, it was asked if hypertonic sucrose and the associated increase in intraendosomal osmotic pressure would also prevent ILV invagination and ultimately translocase activity. Wortmannin’s mode of activity has been reported to prevent material from accessing late, multivesicular endosomes, limiting the ability of the translocase to function [11,16].

## 2. Materials and Methods

### 2.1. Materials

The LSM880 confocal microscope with an Airyscan unit was from Carl Zeiss Ltd., (Oberkochen, Germany), the Triathler scintillation counter was from Hydex (Turku, Finland), and the Multiskan™ FC Microplate Spectrophotometer was from ThermoFisher Scientific (Horsham, UK). Nucleofection was performed using a Nucleofector 2b (Lonza, Basel, Switzerland). RT-qPCR was performed using a QuantStudio 3 system from Applied Biosystems (ThermoFisher Scientific, Horsham, UK). NIH Image J (version 1.54g) [20] was used to perform densitometry after the digitisation of exposed X-ray films. MCF7 STAT3 downregulation Western blots were also captured using a ChemiDocMP imaging system (BioRad Ltd., Watford, UK). Lipofection reagents were from Invitrogen (ThermoFisher Scientific, Horsham, UK). Wortmannin, and wheatgerm agglutinin were from ThermoFisher (Invotrogen, Paisley, UK). All other laboratory reagents were from Sigma Chemical Company (Dorset, UK) unless otherwise stated. Bacterial lysates were generated using a French Press (ThermoFisher Scientific, Horsham, UK) set at 1500 psi. The source and details of the siRNA used for radiolabelling (Table 1), gene silencing (Table 2), TaqMan Probes (Table 2) and antibodies used are given (Table 3).

The details of the cells used and their culture conditions are given (Table 4). The oligonucleotides used for sequencing the transgene inserted into the HEK293 SC008 cells are also detailed (Tables S1–S4).

### 2.2. Methods

Cell culture: Cells were cultured as described (Table 4) under standard incubation conditions (i.e., at 37 °C in a humidified atmosphere containing 5% (*v*/*v*) CO_2_). Dulbecco’s minimal essential medium (D-MEM), Eagle’s minimum essential media (E-MEM), Roswell Park Memorial Institute (RPMI)1640 media, foetal bovine serum (FBS), 100× penicillin–streptomycin-glutamine, 100× nonessential amino acid (NEAA), and blasticidin S HCl (10 mg/mL) were from Gibco (ThermoFisher Scientific, Paisley, UK). Cells were maintained in T75 culture flasks with vented caps and passaged through subconfluent twice a week. For subcellular fractionation, cells were cultured in T175 dishes and grown until 90% confluency using 15 mL of complete media. When needed, cells were washed using sterile phosphate-buffered saline (PBS).

Recombinant protein production: Recombinant PA83, LFn-PKR, and LFn-DTA were produced as previously described [4,8,12] and characterised by Western immunoblotting and Coomassie staining. After dialysis, the proteins were gently filter-sterilised using a 0.2 µ filter and stored at −20 °C as 1 mL aliquots in PBS. The PA preparations were also filter-sterilised and stored at a concentration not exceeding 2 mg/mL at −20 °C.

X-gal colorimetric reporting system: HEK-293 SC008 cells stably expressing green fluorescent protein (GFP) fused-in-frame with beta-galactosidase (LacZ) and bicistronic red fluorescent protein (RFP) were mobilised using sterile trypsin EDTA (TE) buffer (Gibco, ThermoFisher, Paisley, UK) and used to seed a 6-well plate at a cell density of 1 × 10^6^/well, then incubated overnight in complete media. After incubation, the cells were treated with either media (control), WGA (ThermoFisher Scientific, Paisley UK), sucrose or wortmannin at the stated concentrations for 24 h. Cells were then incubated with 50 µg/mL PA83 and 50 µg/mL LFn-PKR, which had been preincubated with siRNA directed against GFP for 5 min at room temperature to reach the stated siRNA concentration. Subsequently, the cells were incubated at 37 °C for 48 h for each treatment. After the indicated incubation period, media from the 6-well plates (Sigma-Aldrich, Dorset, UK) were discarded, 500 µL of RIPA buffer (lysis buffer) (Sigma Aldrich, Dorset, UK) was added to each well, and the plate was incubated on ice for 15 min. The RIPA buffer was aspirated in each well to facilitate cell disruption and added to a labelled sterile 1.5 mL Eppendorf tube, which was centrifuged for 10 min at 21,000× *g* and 4 °C. The supernatant was decanted into a fresh, sterile Eppendorf tube, and (3 × 10 µL) aliquots were used to estimate protein concentration using a bicinchoninic acid (BCA) assay kit (Sigma, Dorset, UK), following the manufacturer’s instructions. Following this, 400 µL of supernatant was added to 12 µL of X-gal (50 mM in DMSO), at which point aliquots (100 µL) were added to a 96-well plate. Hydrolysis of X-gal was then measured at 37 °C at an absorbance of 620 nm over a total time of 6 h 30 min, taking a reading every 15 min. At each instance, the point of maximum divergence from the control was used to calculate the degree of knockdown.

LFn-DTA cytotoxicity assay: Toxicity profiles used to determine the optimal amount of PA and LFn-DTA were measured. Here, a 16 mg/mL stock of LFn-DTA was diluted to the stated concentration using complete cell culture media (initially as 2x stock solutions to accommodate the displacement of a second 2x concentration of PA solution). HeLa cells were seeded at 1 × 10^4^ cells/well in a 96-well TC-treated sterile plate and left under standard TC incubation conditions for 24 h. The (2x) PA solution was prepared at twice the stated concentrations. The media from the HeLa cells was removed and replaced with either 50 µL of the (2x LFn-DTA) solution and then 50 µL of the (2x PA) solution, or by substituting one or both reagents with complete media to generate appropriate controls, i.e., PA with no LFn-DTA, LFn-DTA with no PA, and cells with no LFn-DTA or PA. The cells were then incubated for 45 h prior to the addition of 20 µL of sterile 5 mg/mL 3-4,5 dimethyl-thiazol-2,5 diphenyl tetrazolium bromide (MTT) in PBS for 3 h under standard incubation conditions. The media were then removed, and 100 µL of dimethyl sulfoxide (DMSO) was added to each well. After 20 min, the OD_560_ was recorded. Absorbance values were imported into Excel (Microsoft Corporation, Redmond, WA, USA), and the data were expressed as cell viability (%) relative to an untreated control. For the inhibition studies, 6-well plates were used, and the cell seeding density was adjusted to 1 × 10^6^cells/well. The PA::LFn-DTA complex was prepared at the stated concentrations of PA, LFn-DTA, and inhibitor in complete cell culture media to a total volume of 2 mL, which was added to each well of cells where stated. After the stated incubation period, cell viability was assessed through the addition of 20 µL of 5 mg/mL sterile MTT diluted in PBS. Cells were then incubated for 3 h, the media were removed, and 100 µL of DMSO was added. The plate was then incubated for 30 min at room temperature, and readings were taken at 560 nm using a Multiscan EX Microplate photometer (ThermoLifeSciences, Essex, UK).

Confocal imaging: HeLa cells were treated with the stated concentrations of the listed inhibitors, which were chased into a late endocytic compartment. This was achieved by adding WGA-conjugated Alexa Fluor^®^488 (WGA-A488) or Texas Red (WGA-TxR) dissolved in PBS and filter-sterilised using a 0.2 µ filter to a previously established monolayer of cells grown on a coverslip in a 6-well culture plate at a density of 1 × 10^6^ cells/well. This “pulse” of Alexa Fluor 488- or Texas Red-labelled WGA (Invitrogen, ThermoFisher Scientific, Paisley, UK) was incubated with cells for 4 h at 37 °C in complete media. This was then washed with PBS, and cells were then bathed in complete media. Following an 18 h incubation (“chase”) period, the cell monolayer was washed with PBS, and the cells were fixed. Fixation was performed by adding freshly prepared 2% (*w*/*v*) paraformaldehyde (PAF) in PBS to the cell monolayer. After three washes in PBS, Triton-X-100 permeabilization buffer also in PBS was added containing diamidino-2-phenylindole (DAPI) at a final concentration of 100 ng/mL. After three washes in PBS, 1 mL of blocking buffer (PBS containing 2% (*v*/*v*) FBS) was added to each well and left for 1 h. The coverslips with fixed cells were then mounted onto microscope slides in PBS containing 1% (*w*/*v*) *n*-propyl gallate and 50% (*v*/*v*) glycerol before being sealed (using nail varnish) and imaged using a confocal LSM880. Live cell imaging of the HEK293 cells was achieved using glass-bottomed cell culture plates (ThermoFisher Scientific, Paisley, UK) and imaging the cells in complete media without phenol red. To this end, 5% (*v*/*v*) CO_2_ was passed over the open culture dishes, and images were acquired at the designated time using a LSM880 confocal microscope with its environmental chamber humidified and set to 37 °C. The environmental chamber was set up 4 h prior to imaging to allow all components to reach equilibrium and to prevent drift in the *z*-axis.

Radiolabelling siRNA with [γ-^32^P] ATP: All local and national training and safety guidelines were observed when receiving, handling, and disposing of ^32^P. Radiolabelling of siRNA was achieved using [γ-^32^P]-3000 Ci/mmol 10 mCi/mL EasyTide ATP (PerkinElmer, Coventry, UK) and a 5’ end-labelling kit (Promega, Hampshire, UK) and following the manufacturer’s recommendations. Briefly, 24 µL of a 20 mM siRNA solution was treated with calf intestinal alkaline phosphatase (CIP) to remove the 5’ phosphate. After 30 min, at 37 °C, a further 1 µL of CIP was added to the reaction, and it was incubated for a further 30 min at 37 °C. The preparation was heated to denature the CIP, and the dephosphorylated siRNA was recovered by solvent extraction and isopropanol precipitation. This was performed by first adding an equal volume of phenol/chloroform and recovering the aqueous phase by centrifugation at 12,000× *g* for 1 min at room temperature, then adding an equal volume of phenol chloroform/isoamyl alcohol before recovering the aqueous phase as before. The siRNA was then recovered by isopropanol precipitation. The siRNA pellet was diluted into 24 µL of DEPC-treated water, and the 5’ phosphate was replaced with a ^32^P atom retrieved from 8 µL of [γ-^32^P]-3000 Ci/mmol 10 mCi/mL EasyTide ATP, using T4 polynucleotide kinase. The reaction was stopped using EDTA, and the ^32^P-labelled siRNA was recovered by solvent extraction and isopropanol precipitation as before, leaving unincorporated radioactivity in the supernatant, which was removed and safely disposed of. The pellet was then dissolved in 24 µL of DEPC-treated water. Specific activity was assessed by running a known volume of the ^32^P-labelled siRNA through a 15% (*w*/*v*) native PAGE gel, staining it with gel red, and using densitometry to estimate the mass relative to a selection of cold siRNA standards. Free ^32^P was quantified by placing 10 µL of a 1:100 dilution of the ^32^P-labelled siRNA on to a 2 cm square of Hybond N + membrane, washing it with sodium phosphate buffer, comparing the emission (CPM) from the membrane with the input, and recording the emissions from the washing buffer with the input and those remaining on the membrane. Scintillation counting was performed over 60 s using a Triathler scintillation counter (Hydex, Turku, Finland) and using 2 mL of scintillation fluid (Flo-Scint II, PerkinElmer, Coventry, UK).

Subcellular fractionation. Subcellular fractionation was performed by differential centrifugation using a previously published protocol [4,21,22]. Briefly, cells were grown to 90% confluence in 3xT175 dishes and were transfected in a final volume of 8 mL/dish using serum-free media. Transfections were performed as previously stated [4] using 50 µg/mL of PA, 50 µg/mL of LFn-PKR, and the indicated amount of siRNA. Where appropriate, the labelled siRNA (5.4 × 10^8^ CPM for each experiment) was diluted with cold (non-labelled) siRNA. The siRNA was allowed to hybridise to the LFn-PKR for 5 min in half of the final volume of serum-free media before being added to the PA solution, and the volume was then adjusted to the required amount using serum-free media. After the removal of the existing complete media, the cells were washed 3x using sterile PBS, which was also removed prior to the transfection media being added to the cell culture dishes. At the designated time, the cell culture media were removed, the cells were washed 3x in PBS, which was removed, and the excess was blotted from the edge of the culture dish. Next, 200 µL of HES buffer (20 mM HEPES pH 7.4, 250 mM sucrose, and 1 mM EDTA) was added to the first plate. The cells were mobilised using a rubber policeman, and the cell suspension was transferred to the next, blotted culture dish. This process was repeated, and the cells were transferred to a 1.5 mL Eppendorf tube. A further 200 µL of HES buffer was used to wash the plates, and the two cell suspensions were combined. Cells were then lysed by rapid passage through a 21-gauge needle (10x) and subject to 600× *g* centrifugation for 2 min at 4 °C. Then, 50% of the supernatant was removed and kept on ice. The other 50% was used to resuspend the pellet, which was passed an additional 10x through a 21-gauge needle. The preparation was subject to 600× *g* centrifugation as before, and the supernatants were pooled. The volume of this lysate was recorded, and 50 µL was removed, frozen, and labelled as lysate. The remaining lysate was subject to 1500× *g* centrifugation for 2 min at 4 °C. The supernatant was removed, the volume was recorded, and 50 µL was removed, labelled, and frozen after being labelled as post-nuclear supernatant (PNS). The pellet was resuspended in 100 µL of HES buffer, labelled as the nuclear fraction, and frozen. The remaining PNS was then subject to 200,000× *g* centrifugation for 60 min at 4 °C and separated into a supernatant (cytosol fraction) and pellet (membrane fraction), which was resuspended in 100 µL of HES buffer and frozen. The following day, the frozen fractions were analysed for protein concentration, and the radionucleotide emissions were recorded for a specific volume. Immunoblotting was undertaken to determine the level of enrichment of control (EEA1), membrane (TfR), and cytosol (LDH) markers, as described. The total amount of protein in the cell lysate was calculated, as were the proportions in the nuclear, membrane, and cytosol fractions. This was then used to calculate the distribution and mass (from the specific activity) of siRNA in each compartment. Previously, leakage from the membrane fraction was controlled for [4], where translocation out of the membrane fraction was shown to be PA-, temperature-, and time-dependent. This observation agrees with our previous work undertaking subcellular fractionation, where lysosomal rupture was only observed after using a polytron cell disrupter rather than a gentler form of lysis and disruption such as a Dounce homogeniser [22].

Western immunoblotting: Immunoblotting was performed as previously described [4], using the antibodies listed (Table 3). Approximately 50 µg of PNS per well was run, and, after separation on 12% (*w*/*v*) acrylamide-resolving gel at 200 v for 45 min, samples were transferred onto a 0.2µ nitrocellulose membrane at 400 mA for 120 min. This blot was then washed in PBS containing 0.01% (*v*/*v*) TWEEN 20, (PBS-T) and blocked by incubation in PBS-T containing 5% (*w*/*v*) non-fat dried milk for 60 min at 37 °C. Following this, the blot was incubated on a shaking platform with the primary antibody (Table 3) diluted in PBS-T containing 5% (*w*/*v*) non-fat dried milk for 60 min at 37 °C. After three × 5 min washes in PBS-T, the blot was incubated with the secondary antibody (Table 3) also diluted in PBS-T containing 5% (*w*/*v*) non-fat dried milk for 60 min at 37 °C. The blot was then washed twice for 5 min (each) in PBS-T and once more for 5 min in PBS before being used to expose X-ray film (Scientific Laboratory Supplies Ltd., Notts, UK) in the presence of (picostable) ECL reagent (ThermoFisher Scientific, Paisley, UK) used in accordance with the manufacturer’s instructions. X-ray film was exposed for times varying between 5 s and 24 h depending on the signal-to-noise ratio and signal strength. Exposed films were developed using an Ecomax X-ray film automatic processor (Photon Imaging Systems, Swindon, UK) following the manufacturer’s instructions. In the case of the anti-STAT3 antibody, the blots were blocked in Tris-buffered saline (50 mM Tris, 150 mM sodium chloride, pH 7.6) containing 0.1% (*v*/*v*) TWEEN 20 and 5% (*w*/*v*) BSA. Blots were washed each time in TBS-TWEEN 20, and primary hybridisation was performed at 4 °C overnight under shaking conditions containing TBS 0.1% (*v*/*v*) TWEEN 20, and 5% (*w*/*v*) BSA. Subsequent repartitions were imaged using a ChemiDocMP system (BioRad Ltd., Watford, UK) following the manufacturer’s recommendations.

Data handling: Data from MTT assays were exported from the Scanit software (6.0) (ThermoFisher Scientific, Paisley, UK) into Excel version 16.103.3, (Microsoft, Reading, UK). Here, the mean and standard deviation (SD) for each treatment were found, and the control (%) was calculated by dividing a given average or SD by 1/100th (i.e., 1%) of the non-treated control’s average reading. These data were then moved into Prism (v10.0) software (GraphPad by Dotmatics, CA, USA) and used to generate a graphical representation of the data. Appropriate statistical analysis was also performed using Prism as stipulated in Section 3. For subcellular distribution experiments, the specific activity of each ^32^P-labelled siRNA preparation was defined as described and used to calculate (allowing for radioactive decay with a half-life of 14.3 days) siRNA mass at a given time within a specific compartment. Knowing the subcellular compartment protein concentrations allowed for the calculation of total compartmental volumes, including the amounts removed to read protein concentration and CPS. The results were expressed as the proportion (%) of total cell associated radioactivity allowing mass/compartment to be calculated. As the input number of cells was also known, an estimate (as a small proportion of cells did not lyse and were removed from the equation and the effect of the treatment on cell doubling time was not established) of siRNA mass/compartment/cell in relation to pharmacological activity was also undertaken. Statistical comparisons were made using an ordinary one-way ANOVA test (Dunnett’s T3 multiple-comparison test) at every instance except for Figure S1c, for which an unpaired *t*-test was used.

## 3. Results

**Figure 1:**
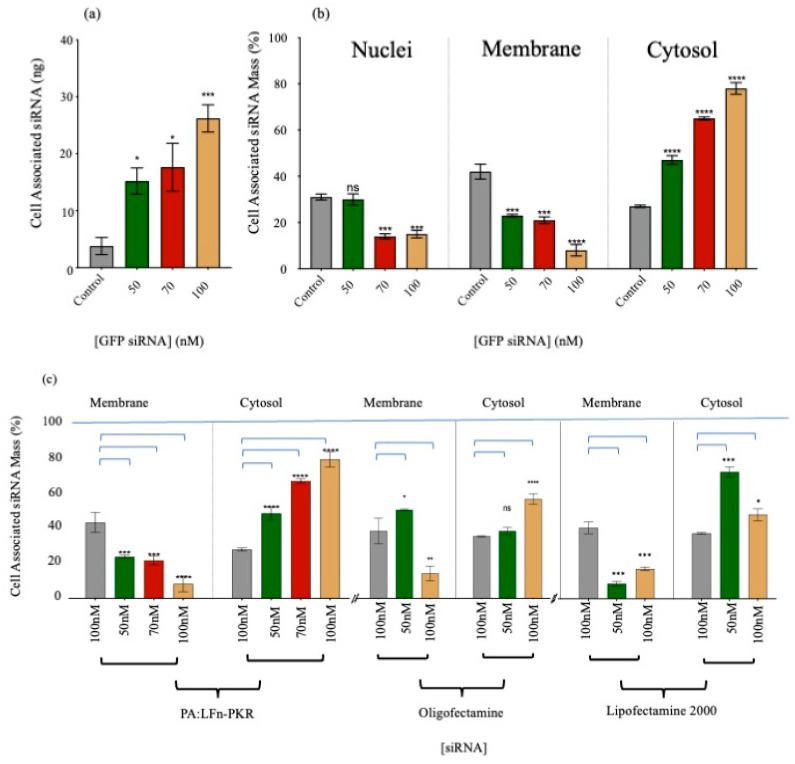
Subcellular ^32^P-labelled siRNA delivery. (**a**) HEK293 SC008 cell-associated ^32^P-labelled siRNA (*n* = 3 ± SD). (**b**) Subcellular distribution of GFP-specific, ^32^P-labelled siRNA delivered using PA::LFn-PKR to HEK293 SC008 cells after 24 h (*n* = 3 ± SD). The grey bar labelled control is siRNA with no delivery system. (**b**) Subcellular distribution of GFP-specific, ^32^P-labelled siRNA delivered using PA::LFn-PKR to HEK293 SC008 cells after 24 h (*n* = 3 ± SD). The grey bar is a control with no delivery system and only the indicated amount of siRNA. The transfection protocol was carried out as previously described [4], and the subcellular fractionation was performed by separating the whole cell lysate into nuclei and PNS by sedimentation at 1500× *g* for 2 min at 4 °C and then separating the PNS into membrane and crude cytosol fractions by sedimentation at 200,000× *g* for 60 min at 4 °C. For context, cell-associated ^32^P was also documented at each dose of siRNA, as this helps explain the non-specific signal distribution at lower siRNA doses. Here, the control was no PA83 or LFn-PKR. (**c**) Intracellular ^32^P-labelled siRNA distribution 24 h after transfection in MCF-7 cells (*n* = 3 ± SD) was investigated. Here ^32^P-labelled siRNA distribution after transfection with PA83::LFn-PKR, Oligofectamine, and Lipofectamine 2000 using the stated doses of siRNA were shown. The control was no PA83 or LFn-PKR. Statistics: (**a**) Control vs. 50 nM, *p* = 0.0132 *; control vs. 70 nM, *p* = 0.0297 *; control vs. 100 nM, *p* = 0.0003 ***. (**b**) Nuclei: control vs. 70 nM, *p* = 0.0002 ***; control vs. 100 nM, *p* = 0.0004 ***. Membrane: control vs. 50 nM, *p* = 0.0043 ***; control vs. 70 nM, *p* = 0.0045 **; control vs. 100 nM, *p* = 0.0003 ***. Cytosol: control vs. 50 nM, *p* = 0.0067 **; control vs. 70 nM, *p* < 0.0001 ****; control vs. 100 nM, *p* = 0.0007 ***. (**c**) PA::LFn-PKR membrane: control vs. 50 nM, *p* = 0.0007 ***; control vs. 70 nM, *p* = 0.0003 ***; control vs. 100 nM, *p* < 0.0001 ****. PA::LFn-PKR cytosol: control vs. 50 nM, *p* < 0.0001 ****; control vs. 70 nM, *p* < 0.0001 ****; control vs. 100 nM, *p* < 0.0001 ****. Oligofectamine membrane: control vs. 50 nM, *p* = 0.0139 *; control vs. 100 nM, *p* < 0.0016 **. Oligofectamine cytosol: control vs. 100 nM, *p* < 0.0001 ****. Lipofectamine 2000 membrane: control vs. 50 nM, *p* = 0.0006 ***; control vs. 100 nM, *p* = 0.0003 ***. Lipofectamine 2000 cytosol: control vs. 50 nM, *p* = 0.0001 ***; control vs. 100 nM, *p* = 0.0448 *.

**Figure 2:**
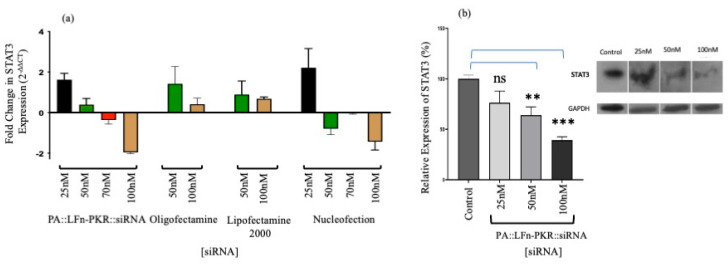
Characterisation of gene silencing. (**a**) Anti-STAT3 siRNA activity after 24 h by RTqPCR in MCF7 cells (*n* = 3 ± SD). (**a**) Target knockdown using anti-STAT3 siRNA after 24 h by RT-qPCR in MCF7 cells (*n* = 3 ± SD) after transfection using a variety of methods, including PA83::LFn-PKR, Oligofectamine, Lipofectamine 2000, and Nucleofection using RT-qPCR. (**b**) Insert STAT3 expression in MCF7 cells by immunoblotting (*n* = 9 ± SD) 24 h after PA::LFn-PKR transfection. (**b**) Gene silencing in MCF7 cells using STAT3-specific siRNA and Western blotting to monitor STAT3 expression levels relative to a housekeeper gene (GAPDH). The inset is a representative blot. Statistics: (**b**) Control vs. 50 nM, *p* = 0.0028 **; control vs. 100 nM, *p* = 0.0004 ***.

The aAtx-based drug delivery technology described herein is preliminary, and questions remain regarding its mechanism of action. Indirect evidence of the PA::LFn-PKR-mediated cytosolic delivery of ASOs and siRNA has been published [4]. These data were surprising given the published mode of action of the PA translocase [11]. Consequently, an assay that could quantify and relate direct evidence reporting the subcellular localisation of siRNA to its pharmacological effect was developed (Figure 1 and Figure 2). To this end, both the HEK293 (SC008) and MCF-7 cell lines were used as a reporter system for RNA interference. Then, having gathered direct evidence for siRNA cytosolic translocation, pharmacological data was gathered that could be used to relate the siRNA subcellular distribution to siRNA activity.

To create a phenotypic assay for siRNA activity using the HEK293 (SC008) cells, an understanding of the transgene sequence coding for the target mRNA was needed. To this end, PCR amplicons spanning the transgene were sequenced (Figure S2a and Tables S1–S4), and the nucleotide sequence was then translated into a protein sequence in silico to control for codon optimisation. This was then used to identify the specific GFP, LacZ, and RFP variants present (Figure S2b–e), i.e., emerald GFP (EmGFP) *E. coli* beta-galactosidase chain 1 and the RFP tandem dimeric tomato (TdTom). The transgene sequence was then deposited in Genbank (accession number MH015340.1, https://www.ncbi.nlm.nih.gov/nuccore/1601116110, accessed on 9 October 2025).

Having ascertained the sequence of the transgene, siRNA sequences were identified that could silence the transgenic mRNA transcripts and consequently reduce GFP, LacZ, and RFP expression. This was confirmed using commercial GFP reporter control siRNA after both Nucleofection^®^ and PA::LFn-PKR-mediated transfection (Figure S1a–f), measured using X-gal hydrolysis (Figure S1a,f), Western immunoblotting (Figure S1b), and RT-qPCR (Figure S1c,d). These data offer indirect evidence that PA::LFn-PKR mediated the cytosolic delivery of siRNA, supporting the finding previously published for HeLa, THP-1, and Vero cells [4]. Gene silencing was observed 48 h after Nucleofection^®^ with 25 nM siRNA (29% ± 0.8% expression relative to the control (±SD; *n* = 3, *p* < 0.0001) (Figure S1a) and 48.9% ± 1.5 expression relative to the control (±SD; *n* = 3, *p* = 0.0001)) after treatment with 100 nM siRNA and PA::LFn-PKR (Figure S1b). When RTqPCR was used to monitor gene expression, after delta delta CT analysis (Figure S1c), 0.36 ± 0.13 (±SD; *n* = 3) expression was reported after the Nucleofection of 100 nM of GFP-specific siRNA. When PA and LFn-PKR were used, 0.4 ± 0.07 (±SD, *n* = 9) expression of the control transgene was reported (Figure S1d). Gene knockdown was (qualitatively) visible when the cells were visualised by confocal microscopy after transfection (Figure S1d). Gene silencing relative to a sham-transfected control (Figure S1e (row i)) reports a qualitative reduction in both green and red signals after transfection by Nucleofection^®^ (Figure S1e (row ii)) or PA::LFn-PKR (Figure S1e (row iii)) using identical detector gain settings across samples. This evidence was again observed when enzymatic activity was used to monitor gene expression (Figure S1f). Here, after transfection using PA, LFn-PKR, and 100 nM of GFP siRNA, 79.95 ± 9.78% (±SD, *n* = 3) inhibition was documented.

Direct evidence for PA::LFn-PKR-mediated siRNA cytosolic delivery was also gathered using radiolabelled siRNA (Table 1) and the subsequent subcellular fractionation of two distinct cell lines (HEK293 (SC008) and MCF-7). Scintillation counting the subcellular fractions of treated cells provided direct evidence of the cytosolic delivery of PA::LFn-PKR-mediated ^32^P-labelled siRNA in both cell lines cells (Figure 1a–c). The cell-associated siRNA (Figure 1a) distribution (%) (Figure 1b) provides a relative overview of the enrichment of siRNA within the subcellular compartments and the absolute siRNA mass/compartment. Figure 1b records 78 ± 2% (±SD, *n* = 3) of cell-associated radioactivity to be in the cytosol of HEK293 cells 24 h after being incubated with 100 nM siRNA, delivered using the aATx system. Transfection was performed with the ^32^P-labelled siRNA (GFP reporter control) described in Table 1, using three concentrations of siRNA and a control without PA or LFn-PKR. The distribution of cell-associated radioactivity was reported (Figure 1b), although the control data should be interpreted with care and after considering the varying uptake (mass) associated with the different levels of specific activity (Figure 1a). Here, it is also important to consider that the mass needed for higher concentrations of siRNA was beyond using only labelled siRNA. Consequently, a constant amount of labelled RNA was used and, in each instance, diluted with unlabelled RNA (giving a different specific activity) depending on the dose needed for the experiment. The constant dose used per experiment was 5.4 × 10^8^ CPM.

This observation explains why the control shows a high percentage of siRNA in the cytosol (Figure 1b), although the mass of the siRNA within the cytosol was very low (after a 100 nM dose) (Figure 1b). What was striking was that after transfection with PA::LFn-PKR, there was an increase in cytosolic siRNA even when accounting for the different masses of siRNA internalised as a function of dose (Figure 1a) and that this enrichment was statistically significant (*p* = 0.0007) relative to the control. Representative marker enrichment patterns indicated that the fractionation was successful (Figure S3), as there was no detectable cytosolic marker (LDH) visible in the membrane fraction and no membrane marker (TfR) visible in the cytosolic fraction. The presence of EEA1 in both fractions acted as a loading control for both HeLa and MCF7 cells.

Figure 1 also relates the translocation of ^32^P-labelled siRNA mass and, in turn, the number of siRNA molecules into the cytosol (Figure 1a–c and Table 1) to RT-qPCR (Figure 2a) and Western analysis (Figure 2b) profiles. Consequently, it was possible to determine that a dose of 50 nM of siRNA reduced STAT3 gene expression to 53.2 ± 8.12% (±SD; *n* = 3) of the control (Figure 2b), delivering approximately 1.3 × 10^13^ siRNA molecules to the cytosol/cell. A dose of 100 nM siRNA resulted in a 35.9 ± 14.2% (±SD; *n* = 3) expression of STAT3 (Figure 2b) after 3.9 × 10^13^ siRNA molecules were detected in the cytosol.

A comparison of the efficiency of cytosolic siRNA delivery by various transfection systems was also undertaken. Figure 1c documents the cytosolic enrichment of ^32^P-labelled siRNA, and Figure 2a documents siRNA activity by RTq-PCR and Western immunoblotting. Approximately 77 ± 2.5% (±SD; *n* = 3) of the cell-associated siRNA was documented in the cytosol after transfection using PA::LFn-PKR and 100 nM ^32^P-labelled siRNA, compared to 56 ± 1.7% (±SD; *n* = 3) after the transfection of 100 nM ^32^P-labelled siRNA using Oligofectamine or 45 ± 3.2% (±SD; *n* = 3) using Lipofectamine 2000. Figure 2b relates this distribution to a reduction in mRNA level. Significant knockdown was observed after transfection with PA::LFn-PKR and by Nucleofection. These data were further verified by Western analysis, where a reduction in target gene expression was documented relative to a housekeeper gene (GAPDH) after using 100 nM siRNA with PA::LFn-PKR (Figure 2b). Here, treatment with 100 mM siRNA delivered using PA::LFn-PKR resulted in a 36 ± 14.17% (±SD, *n* = 3) STAT3 gene expression. Representative subcellular marker enrichment across the PNS, cytosol, and membrane fractions demonstrated that there was no detectable cytosol marker in the membrane fraction and no detectable membrane marker in the cytosol fraction (Figure S3a,b).

**Figure 3:**
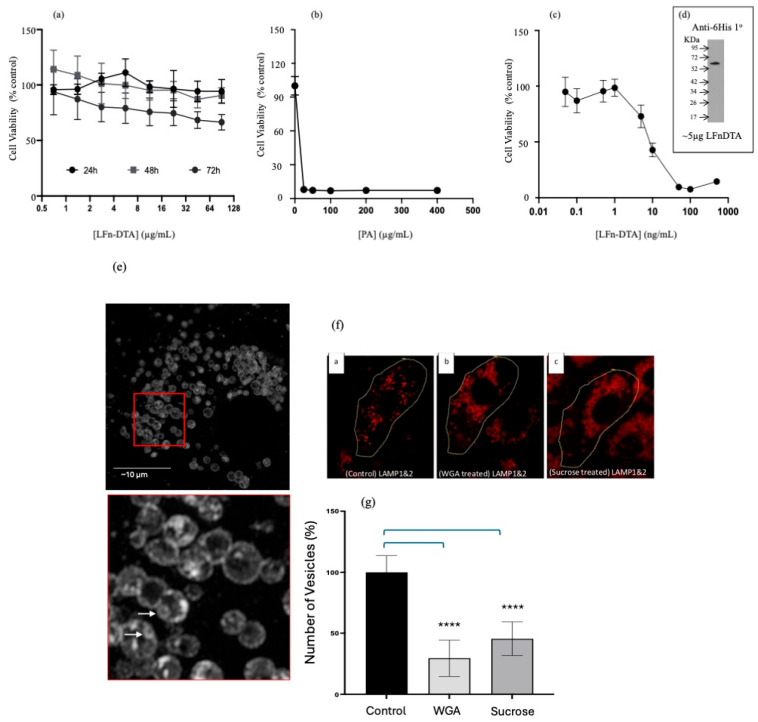
Assaying aATx cytosolic delivery via ILVs. (**a**). HeLa viability after treatment with LFn-DTA over time (±SEM; *n* = 6) (**a**) Toxicity profile of LFn-DTA over time in HeLa cells without PA. (**b**). HeLa viability 48 h after treatment with PA83 and LFn-DTA (10 µg/mL) (±SEM; *n* = 6). (**b**) Toxicity profile of LFn-DTA at a concentration of 10 µg/mL and varying amounts of PA after 48 h. (**c**). HeLa viability 48 h after treatment with PA83 (5 µg/mL) and LFn-DTA (±SEM; *n* = 6) (**c**) Toxicity profile of varying amounts of LFn-DTA with 5 µg/mL of PA83. The inset is a Western blot of the recombinant LFn-DTA used after detection with a 6His-specific monoclonal primary antibody and HRP-labelled anti-mouse secondary antibody (**d**). (**e**) Super-resolution confocal images of Vero cells immunostained using anti-LAMP1 and 2 followed by a Texas-Red-labelled anti-mouse secondary antibody. The red square has been further magnified below and ILVs are signified with arrows. (**e**) Intraluminal vesicles within the late endocytic (LAMP + ve) compartment of Vero cells. (**f**) ILVs within LAMP-positive structures (LEs) after treatment with WGA or sucrose labelled a, b, and c for different treatment conditions. (**f**) Reduction in ILVs in Vero cells after treatment with WGA or sucrose when examined using imunostained cells by confocal microscopy. This disparity is quantified in (**g**). (**g**) Quantification of ILVs within LEs (*n* = 10 ± SD) after treatment with either WGA or sucrose, Statistics: (**g**) control vs. WGA, *p* < 0.0001 ****, control vs. sucrose, *p* < 0.0001 ****.

Having ascertained that siRNA was translocated into the cytosol using both direct and indirect assays within multiple cell lines, the role of ILVs during the PA-mediated transfection of siRNA was investigated. Given that after the hydrolysis of PA83 to PA63 needed to form homo-heptamers or homo-octamers and the insertion of the PA63 pre-pore into the membrane to form the PA63 pore, the possibility that these events destabilised the limiting membrane of endocytic vesicles, rather than the PA multimer acting as a translocase, was investigated. This was carried out using two different types of translocase substrates. The first mimicked the wild-type translocation of LF and required the use of an LF truncation (i.e., the first 255 N-terminal amino acids) fused-in-frame to diphtheria toxin a chain (DTA). Figure 3a assayed the toxicity of LFn-DTA (without PA) over time and over the microgram concentration range. Over a microgram concentration range, little variance from the control population viability was documented 24, 48, and 72 h post-treatment. Figure 3b reports the effect of adding various concentrations of PA protein to sub-lethal concentrations of LFn-DTA. The presence of the PA reduced cell viability to below 10% of the control population at every PA concentration measured, down to a PA concentration of 25 µg/mL. When this PA concentration was reduced further to 5 µg/mL and the concentration of LFn-DTA was reduced to 50 ng/mL (Figure 3c), less than 10% cell viability was also documented. The LFn-DTA protein was characterised by Western immunodetection using a 6His-specific antibody, as shown (Figure 3d). This was due to the lack of a reliable LFn-specific antibody.

The next step is characterising the effect of WGA and hypertonic sucrose on ILV formation (Figure 3e). Here, Vero cells were observed after the cells were fixed in cold methanol and immunolabelled with antibodies specific to lysosome-associated membrane proteins (LAMP) 1 and 2. ILVs are clearly visible in the inset as membrane-inside-membrane delineated vesicles, and specific examples are marked with arrows (Figure 3d). Figure 3f gives a qualitative indication of the effects of both sucrose and WGA on ILV formation, and this was quantified (Figure 3f).

To quantify the number of ILVs, ImageJ (version 1.54g) was used to analyse cell populations, which were visualised using the LSM880 confocal microscope (and an appropriate threshold was set). A free-hand outline was drawn around the control (no treatment), WGA-treated, and sucrose-treated cells. The “Analyse particle” command was used to distinguish individual LAMP1- and 2-stained vesicles and classify each as a discrete entity. Vesicles were outlined, counted, and measured. The results were represented as a percentage relative to the untreated control (Figure 3f). WGA-treated cells showed a reduction in ILV formation of 28.5 ± 14.8% (±SD; *n* = 10), while sucrose-treated cells demonstrated a decrease in ILV formation of 45.4 ± 13.7 (±SD; *n* = 10). Both calculations were compared to the untreated cells stained with LAMP1- and 2-specific antibodies at 100 ± 10.0% (±SD; *n* = 10).

**Figure 4:**
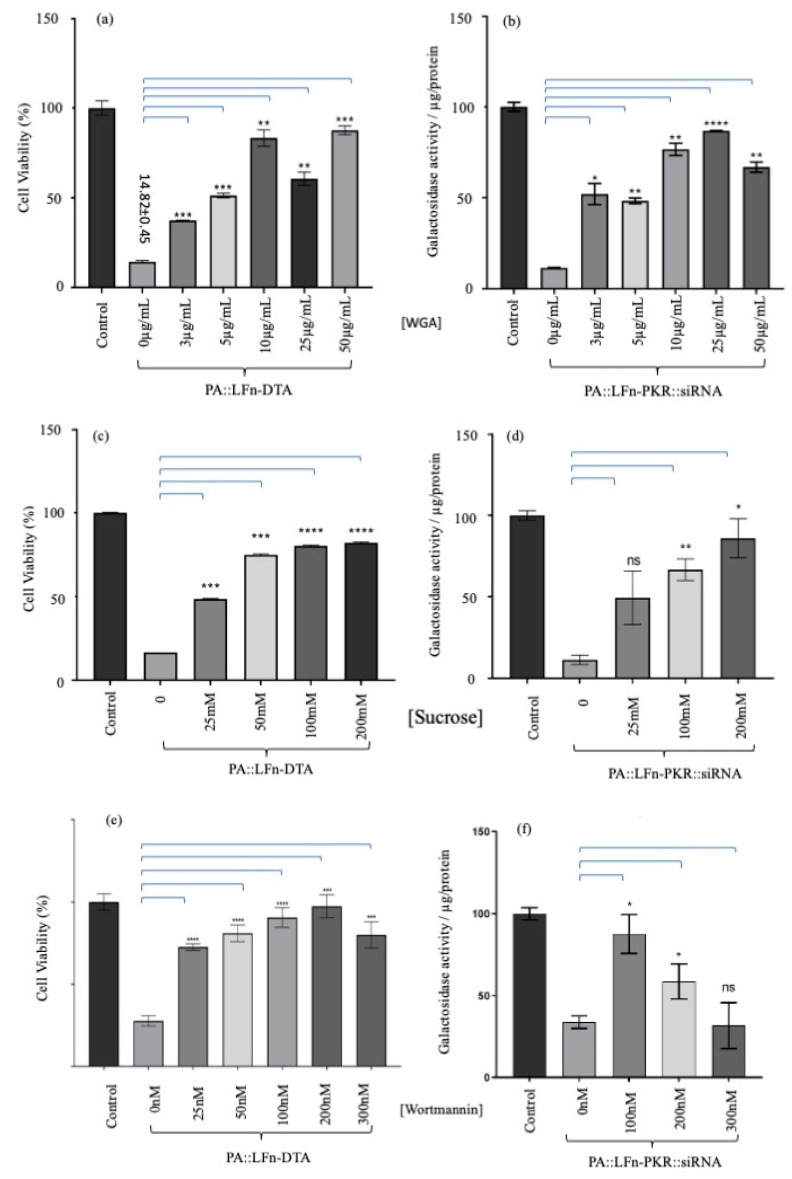
Assaying the effect of ILV inhibitors on aATx-mediated cytosolic delivery. Evaluation of different concentrations of WGA on PA translocase activity using (**a**) LFn-DTA or (**b**) LFn-PKR::siRNA as cargo (*n* = 3 ± SD). (**a**) The effect of increasing concentrations of WGA on the PA::LFn-DTA intoxication of HEK293 SC008 cells. As increasing amounts of WGA were added to the assay, a significant a reduction in LFn-DTA-mediated intoxication was documented. (**b**) documents statistically lower levels of PA::LFn-PKR::siRNA activity in the presence of WGA. When a different inhibitor of ILV biogenesis was used (hypotonic sucrose), a similar result was observed. The evaluation of the effect of different concentrations of sucrose on PA translocase activity was carried out using (**c**) LFn-DTA or (**d**) LFn-PKR::siRNA as cargo (*n* = 3 ± SD). Further, (**c**) documents a statistically significant reduction in PA83::LFn-DTA-mediated toxicity when increasing concentrations of sucrose are incubated with the cells, whereas (**d**) records a sucrose concentration-dependent reduction in siRNA activity. The evaluation of the effect of different concentrations of wortmannin on PA translocase activity was carried out using (**e**) LFn-DTA or (**f**) LFn-PKR::siRNA as cargo (*n* = 3 ± SD). Here, a more general inhibitor of endocytosis (wortmannin) was also examined and incubated with cells in the presence of PA83 and LFn-DTA. As might be predicted, there was also a statistically significant reduction in PA83::LFn-DTA intoxication after treatment with increasing concentrations of wortmannin. (**f**) The effect of wortmannin on PA83::LFn-PKR::siRNA gene silencing activity. (**e**) A reduction in gene silencing was recorded at 100 nM and 200 nM concentrations of wortmannin. Statistics: LFn-DTA assay: 0 µg WGA vs. 3 µg WGA, *p* = 0.0003 ***; 5 µg WGA, *p* = 0.0014 **; 10 µg WGA, *p* = 0.0029 **; 25 µg WGA, *p* = 0.0041 **; 50 µg WGA, *p* = 0.0007 ***. Transfection assay: 0 µg WGA vs. 3 µg WGA, *p* = 0.0216 *; 5 µg WGA, *p* = 0.0031 **; 10 µg WGA, *p* = 0.0054 **; 25 µg WGA, *p* < 0.00001 ****; 50 µg WGA, *p* = 0.0044 **. Sucrose LFn-DTA assay: 0 mM sucrose vs. 25 mM sucrose, *p* = 0.0001 ***; 50 mM sucrose, *p* = 0.0003 ***;100 mM sucrose, *p* < 0.0001 ****; 200 mM sucrose, *p* < 0.0001 ****. Sucrose transfection assay: 0 mM sucrose vs. 100 mM sucrose, *p* = 0.0108 *; 200 mM sucrose, *p* = 0.0019 **. Wortmannin assay: 0 nM wortmannin vs. 25 nM wortmannin, *p* = 0.0001 ***; 50 nM wortmannin, *p* = 0.0012 **; 100 nM wortmannin, *p* < 0001 ****; 200 nM wortmannin, *p* < 0001 ****; 300 nM wortmannin, *p* < 0001 ****. Transfection assay: 0 mM wortmannin vs. 100 nM wortmannin, *p* = 0.0130 *; 200 nM wortmannin, *p* = 0.0181 *.

The effects of WGA, hyperosmotic sucrose, and wortmannin on PA translocation were assayed and documented (Figure 4). Figure 4a,b detail the effects of increasing concentrations of WGA on LFn-DTA or LFn-PKR::siRNA PA translocase activity (respectively). Here, a WGA concentration-dependent increase in translocase inhibition was documented when the activity of both cargo types was examined.

Figure 4a records a cell viability of 14.8 ± 0.45% (±SD, *n* = 3) for the control population when the cells are treated with no WGA and 5 µg/mL PA and 10 µg/mL LFn-DTA. When 10 µg/mL WGA, 5 µg/mL PA, and 10 µg/mL of LFn-DTA were added to the cell culture, a viability of 83.3 ± 3.8% (±SD, *n* = 3) of the control viability was recorded. Figure 4b records the effect of WGA on PA- and LFn-PKR-mediated transfection. Here, 11.5 ± 0.03% (±SD, *n* = 3) expression of LacZ was recorded when uninhibited transfection with PA and LFn-PKR was used. This was in contrast to the 86.7 ± 0.6% (±SD, *n* = 3) expression recorded when 25 µg/mL WGA was added to the cell culture, along with PA::LFn-PKR::siRNA reagent (Figure 4b).

Figure 4c records cell viability after treating the cells with 200 mM sucrose (plus 5 µg/mL of PA83 and 10 µg/mL of LFn-DTA), which resulted in a cell viability of 82.7 ± 0.4 (±SD, *n* = 3). This differs from cells treated with only a similar concentration of PA and LFn-DTA (16.1 ± 0.01% (±SD, *n* = 3) viability) when no sucrose was used. Figure 4d records LacZ expression/µg cell protein. Here, knockdown in the absence of sucrose LacZ expression was recorded as being 11.3 ± 5.1% (±SD, *n* = 3) of the control. After treatment with 200 mM sucrose, expression was recorded to be 86.1 ± 7.76 (±SD, *n* = 3).

When the effects of wortmannin were examined. PA and LFn-DTA at the same concentrations as before left 27.7 ± 1.9% (±SD, *n* = 3) of the control cells alive (Figure 4f). This was in contrast to cells treated with 200 nM wortmannin, which maintained a viability 97.4 ± 2.1% (±SD, *n* = 3) of the control cells when treated with a similar amount of PA and LFn-DTA. Figure 4f records the expression of LacZ in relation to transfection using PA, LFn-DTA, and wortmannin. Here, the control cells subject to siRNA transfection expressed 32.7 ± 4.74% (±SD, *n* = 3) of control lacZ. Under similar transfection conditions and with the addition of 100 nM wortmannin, 85.3 ± 11.7% (±SD, *n* = 3) expression of control LacZ was recorded.

**Figure 5:**
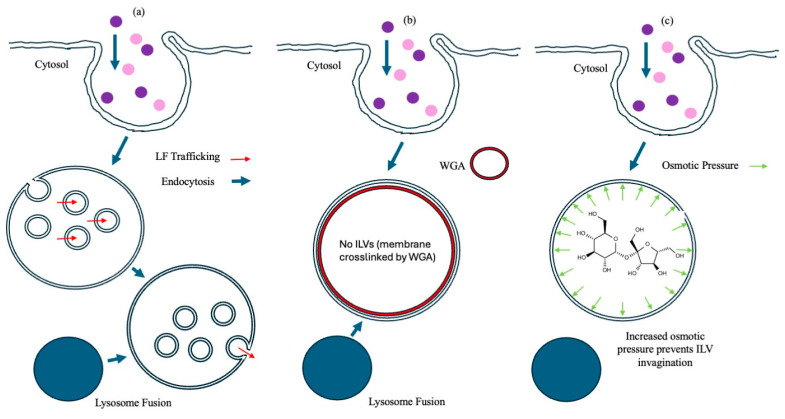
Proposed mode of inhibitor action. This is a cartoon depicting the proposed mode of inhibition of PA pore translocation in the case of no treatment (**a**), (**b**) WGA, and (**c**) hypertonic sucrose.

One interpretation of these data might be that WGA, which has the capacity to crosslink membranes, reinforced the limiting membrane of endosomes, leading to a reduction in the probability of a breach (i.e., reducing endosomolysis). This possibility was countered by increasing the luminal osmotic pressure of the endosomes using hyperosmotic sucrose, which has also been used to reduce ILV formation. The effects of hyperosmotic sucrose on LFn-DTA translocation and LFn-PKR::siRNA translocation were documented (Figure 4c,d), respectively. Rather than a bolus release of translocase cargo indicative of failed endosomal limiting membrane integrity, as might be expected if cargo release simply bypassed the PA pore and was a result of endosomolysis, a sucrose concentration-dependent inhibition of cargo activity was reported. This indicated that the limiting membrane of the endosomes remained intact and that ILV formation was critical for the efficient cytosolic translocation of both LFn-DTA and LFn-PKR::siRNA. Finally, the effects of wortmannin on translocase activity were investigated, and, again, a wortmannin concentration-dependent reduction in cargo activity was reported (Figure 4e,f). This indicates that endocytic fusion events involving the early endosome were also critical to cargo translocation, as might be predicted. Figure 5 captures these data as a cartoon and relates the activity of the translocase cargo to the various ILV inhibitors assayed.

## 4. Discussion

The use of membrane-delineated intermediaries such as an ILV to protect the limiting membrane of a late endocytic structure from rupturing whilst increasing the available surface area of membrane to mediate translocation remains unique to ATx. Toxins such as streptolysin O (SLO) have been found within populations of ILVs. However, this localisation is not intrinsic to the translocation of an A chain into the cytosol but more likely a defence against the unregulated movement of cytosol through a pore within the plasma membrane [23]. SLO ILV localisation is thought to be driven by the ubiquitylation of a cytosolic portion of the toxin, and ubiquitin (Ub) is known to serve as a signalling system that marks the ubiquitinylated target for endolysosomal destruction [24]. In addition to SLO, there is evidence for the ubiquitylation of the cytosolic portion of ATXR1 and 2 [25] but not PA [26]. The pH-driven receptor disassociation and membrane insertion of the PA multimer and the lack of ubiquitylation (the cytosolic portion of PA has been reported to be masked from ubiquitylation by palmitoylation) [26], which may, in part, account for the prolonged residence time of the PA pore with the EEA1-positive (early endosomal) structures previously reported [4], also offer (1) a depot effect for the extended translocation of the material in addition to (2) exosome loading [24,25,27]. Toxins that compromise the limiting membranes of endosomes have also been reported to deliver siRNA to the cytosol, and these include the attenuated diphtheria toxin [28]. This highlights the growing awareness for the need for endosomal escape, although this approach may be self-limiting, as the escape of protons from the endosome lumen may limit subsequent endosomal maturation, transport, and fusion events (as reviewed in [29]). The acidic pH of the endosomal lumen has been reported to drive the Brownian ratcheting [11] or helix compression [30] of PA translocase substrates and facilitate molten globular cargo transition, allowing for passage over the phenylalanine clamp at the base of the PA oligomer vestibule, as well as driving pre-pore-to-pore transition [11]. As most of the PA pore has been reported to be embedded within ILVs [10], there remains the potential to decouple cargo membrane translocation from the membrane-lytic toxicity associated with non-viral delivery technologies such as poly(ethylamine imine) [31] or poly(L-lysine) [32].

After establishing that a pharmacological response was recorded after using PA83, LFn-GAL4, and LFn-PKR to deliver ASOs and siRNA, respectively [4], many questions remained about the role of the cation-selective PA translocase, which has been reported to require cargo molten globular transition to pass through the PA phenylalanine clamp [11]. This clamp has been reported to have a diameter of 6 Å [11], which is too small to allow for the passage of siRNA (a polyanion with a diameter of ~20 Å [33]) and would require the unfolding of the RNA-binding domain of LFn-PKR and the disassociation of the siRNA from the LFn-PKR dimer. This could be interpreted to mean that (1) the PA pore was not only operating as a cation-selective pore but also as an anion-selective pore, (2) the pore could radially dilate, meaning that the phenylalanine clamp’s diameter of 6 Å was that of the pore in a resting state, or (3) the PA pore was not used as a translocase and the insertion of the PA pore transmembrane domain stressed the limiting membrane to the point of endosomolysis. This ultimately begs the question, did the cargo go through the PA translocase into the lumen of an ILV, which then underwent back-fusion with the limiting membrane of the multivesicular body/late endosome?

To understand these observations, this study asked the following question: can siRNA cargo be detected directly in the cytosol as opposed to indirectly, and could this event be quantified? This was addressed using ^32^P-labelled siRNA, which was detected in the nucleic, membrane, and cytosolic fractions of lysed cells subject to increasing relative centrifugal fields. This method of differential centrifugation was adapted from that of DuDuve [34] and previously published [4,22]. Here, the fractionation was characterised by Western immunoblotting. As all the readily detectable cytosolic marker (lactate dehydrogenase (LDH)) [22] was within the cytosolic fraction and not the membrane fraction and the membrane marker (transferrin receptor (TfR)) [35] was detected within the membrane fraction rather than the cytosolic fraction, the fractionation scheme was accepted as generating reliable separation and enrichment of the cytosolic and membrane compartments. Previously, controls were reported showing a requirement for heat and PA for the cytosolic translocation of either LFn-PKR or LFn-GAL4, demonstrating the integrity of vesicles within the membrane compartment [4,22].

Up to 80% of the cell-associated radioactivity (siRNA) was calculated to be in the cytosol after delivery using PA::LFn-PKR, and these data were interpreted as direct evidence of the cytosolic translocation of the siRNA (Figure 1). This conclusion was reinforced by data recording the downregulation of an overexpressed enzyme (LacZ) when examined by (1) assaying beta-galactosidase activity, (2) using RT-qPCR to interrogate mRNA, or (3) by Western immunoblotting to measure protein levels after transfection with both Nucleofection and PA::LFn-PKR (Figure S1).

A quantitative comparison of this transfection system with others characterised in the literature was difficult, as a variety of both in vitro but mainly in vivo datasets have been reported. Further distributions are often reported as a dose (%) rather than a distribution based on a cell-associated label. Whilst there is merit in both approaches, here, in vitro data that denoted the intracellular distribution was adopted rather than a scheme that would also measure cell or organ targeting. Given the efficiency of siRNA capture in an in vitro system and the propensity to manipulate this data by altering the volume of culture fluid and the concentration of label added to the system, it was decided to normalise these data to cell-associated signals, asking the following: what was the subcellular distribution of the captured siRNA? Consequently, comparisons with the conclusions drawn after in vivo studies [36] using colloidal gold as a delivery vehicle and using GalNAc, where cytosolic distributions ranged from 1 to 2% to 0.01% (respectively), remain difficult [37]. Given the proposed mechanism of GalNAc modulating subcellular distribution, this inefficiency is not surprising. Regardless, these papers are still informative and may guide the next stage of this investigation, where in vivo activity may be assessed. Several studies examine the subcellular distribution of nucleic acid drugs in vitro using typically lipid-based delivery vehicles, reporting data with similar distributions to those reported here. The intracellular delivery of FITC-labelled siRNA was documented [38] in KB cells, and 90% was reported in the cytosol using HDL-mimicking nanoparticles. Here, 68 ± 0.3% of the siRNA delivered using Lipofectamine was reported to be cytosolic (Figure 1c). A similar study [39] reported that a lipid-mediated delivery system resulted in 50–100 000 siRNA molecules in the cytosol of HeLa cells using quantitative confocal microscopy. It was reported that Lipofectamine and cell-penetrating peptides resulted in a 60–70% cytosolic distribution of siRNA to the cytosol of ECV-304 cells [40]. The distributions calculated herein, with Lipofectamine being 68 ± 0.3% enriched in the cytosol after a dose of 50 nM (Figure 2a), are comparable with the aforementioned study [38], lending further credibility to the data obtained using PA and LFn-PKR.

Having acquired a substantial body of evidence supporting the idea that PA::LFn-PKR can mediate the cytosolic translocation of siRNA (Figure 1, Figure 2, Figure 4 and Figure S1, [4,8]), the next question addressed the regulation of membrane trafficking responsible for this phenomenon. To this end, two assays were developed to monitor the pharmacological activity of both a well-characterised translocase substrate (LFn-Diphtheria toxin a chain) [8,12] and LFn-PKR::siRNA [4] in the presence of inhibitors of either ILV formation or endocytic fusion events. The first assay used here had been previously characterised and uses the fusion protein LFn-DTA, which, without an exogenous transporter, cannot access its cytosolic target (elongation factor 2a) [12]. In the presence of >5 µg/mL of PA, 10 µg/mL of LFn-DTA is measurably toxic over 48 h when measured using MTT [8].

It was hypothesised that three endocytic inhibitors would impact PA’s ability to act as a translocase by either inhibiting ILV formation or disrupting membrane traffic to the late endosome. WGA is known to crosslink membranes and, at higher concentrations, give rise to expanded late endocytic structures [14]. This observation led to the conjecture that the expansion of vesicle size driven by membrane crosslinking would inhibit ILV formation. Upon microscopic examination of WGA-treated cells, a reduced level of internal membranes within late endocytic structures was documented, supporting this conjecture (Figure 3e,f) and validating the use of these phenomena to examine the role of PA in LFn-PKR::siRNA membrane translocation. Similarly, hyperosmotic sucrose has been reported to also cause expanded late endocytic structures (sucrosomes) with dramatically reduced levels of internal membranes, and this treatment has been found to interfere with the lysosome regeneration cycle [18]. Electron micrographs of sucrose-filled endosomes from NRK cells have also revealed a scarcity of internal membranes (indicative of ILV biogenesis) within late endosomes [17].

When these two inhibitors were used to treat cells, a significant, concentration-dependent reduction in the pharmacological activity of either PA::LFn-DTA or PA::LFn-PKR::siRNA was recorded (Figure 4). This value was similar to the data characterising the effect of the downregulation of ALiX during LF PA translocation [10] and indicates that ILVs form a critical part of the machinery needed for the PA translocase to move LFn-DTA and LFn-PKR::siRNA to the cytosol efficiently. These data suggest the possibility of an endosomolytic effect accounting for translocase cargo release. Given that WGA may reinforce the limiting membrane and possibly reduce the opportunity for PA to become embedded into the membrane, the treatment with hyperosmotic sucrose might be expected to further stress the membrane, enhancing the opportunity for the release of luminal content if membrane integrity was compromised. This opposes the effect the data described here, i.e., the inhibitors reduce pharmacological activity rather than enhance it by further stressing the integrity of the endosomal membrane (Figure 4c,d). Whilst it was possible that hyperosmotic sucrose also inhibited fusion to the LE, this inhibition of fusion was not apparent within populations of WGA-treated cells, where unlabelled gaps in the existing limiting membrane containing fluorescent WGA were documented and indicative of a fresh membrane fusing to the LE already containing WGA [14]. Wortmannin, whilst not directly impacting ILV formation, indicates that endocytic vesicle fusion to early endosomes was also necessary for PA::LFn-DTA and PA::LFn-PKR::siRNA activity.

## 5. Conclusions

Evidence of siRNA translocation across the PA pore was gathered both indirectly and directly by looking at the subcellular distribution of radiolabelled siRNA and by measuring the activity of the siRNA in relation to the expression level of its target. Evidence for the requirement of PA and the involvement of ILVs during this translocation process was also recorded. This makes the probability of an endosomolytic event being responsible for the cytosolic translocation of the siRNA unlikely, framing this first in class delivery technology as unique in its use of an intermediary compartment during the cytosolic delivery of siRNA. 

## 6. Patents

This work relates to patents WO2014203008 and WO2020030923, owned by Greenwich University Enterprises Ltd.

## Figures and Tables

**Table 1:** Characterisation of ^32^P-labelled siRNA (representative values).

siRNA	Specific Activity (Ci/mmol)	Labelled siRNA (ng/mL)	Labelling Efficiency (%)	Free ^32^P (%)
^32^P-Labelled GFP siRNA	1679.786	40.24	65.55	14.82
^32^P-Labelled STAT3 siRNA	2808.233	75.65	79.12	10.1

**Table 2:** siRNA and TaqMan probes.

Reagent	Utility	Catalogue Number	Supplier
GFP Reporter control siRNA	Stealth siRNA	12935145	ThermoFisher (Invitrogen) (Paisley, UK)
STAT3	Silencer siRNA	AM16708	ThermoFisher (Invitrogen) (Paisley, UK)
STAT3 (VIC)	TaqMan Probes set	Hs00374280_m1	ThermoFisher (Invitrogen) (Paisley, UK)
GAPDH (FAM)	TaqMan Probes set	Hs02786624_g1	ThermoFisher (Invitrogen) (Paisley, UK)
High GC scrambled siRNA	Control Stealth siRNA	12935400	ThermoFisher (Invitrogen) (Paisley, UK)

**Table 3:** Antibodies used.

Antigen	Species	Cat No.	Supplier	Dilution
LAMP1	Mouse MAb	H4A3	DHSB * (Iowa City, IA, USA)	1:10 (IF)
LAMP2	Mouse MAb	H4B4	DHSB * (Iowa City, IA, USA)	1:10 (IF)
STAT3	Mouse MAb	ab119352	AbCam (Cambridge, UK)	1:1000 (IB)
GAPDH	Rabbit PAb	Ab2609746	AbCam (Cambridge, UK)	1:5000 (IB)
TfR	Mouse MAb	612124	BD Bioscience (Berkshire UK)	1:200 (IB)
EEA1	Mouse MAb	E41120	BD Bioscience (Berkshire UK)	1:1000 (IB)
LDH	Mouse MAb	L7106	Sigma (Dorset, UK)	1:200 (IB)
6His	Mouse MAb	631212	ClonTech Ltd. (Oxford, UK)	1:5000 (IB)
RFP	Mouse MAb	6G6	Chromotech (Stockport, UK)	1:500 (IB)
GFP	Rabbit PAb	SAB4301138	Sigma (Dorset, UK)	1:1000 (IB)
Anti-Rabbit HRP conjugate	Donkey	NA934	GE Healthcare (Hatfield, UK)	1:1000 (IB)
Anti-Mouse HRP conjugate	Sheep	NA931	GE Healthcare (Hatfield, UK)	1:1000 (IB)
Anti-Rabbit Texas Red conjugate	Goat	T-2767	ThermoFisher (Invitrogen) (Paisley, UK)	1:200 (IF)
Anti-Mouse Texas Red conjugate	Goat	T-862	ThermoFisher (Invitrogen) (Paisley, UK)	1:200 (IF)
Anti-Rabbit Alexa fluor 488 conjugate	Goat	A-11008	ThermoFisher (Invitrogen) (Paisley, UK)	1:200 (IF)
Anti-Mouse Alexa fluor 488 conjugate	Goat	A-11001	ThermoFisher (Invitrogen) (Paisley, UK)	1:200 (IF)

* Developmental Studies Hybridoma Bank at the University of Iowa, Iowa City, IA, USA. IB = Immunoblotting, IF = Immunofluorescence.

**Table 4:** Cells and culture conditions.

Cells	Transgene	Supplier	Cat No	Culture Conditions	Media
HEK293	EmGFP-LacZ::RFP	ASMBio (Abingdon, UK)	SC008	Split 1:20 twice a week	Dulbecco’s Modified Eagle Medium + 10% (*v/v*) Foetal Bovine Serum, 10 µg/mL blastocydin S, 1× penicillin–streptomycin-glutamine,1× nonessential amino acids
HeLa	-	American Type Culture Collection (VA, USA)	CCL2	Split 1:20 twice a week	Eagle’s Minimum Essential Medium + 10% (*v/v*) Foetal Bovine Serum, 1× penicillin–streptomycin-glutamine,1× nonessential amino acid
MCF-7	-	American Type Culture Collection (VA, USA)	HTB-22	Split 1:3 twice a week	Eagle’s Minimum Essential Medium + 10% (*v/v*) Foetal Bovine Serum, 1× penicillin–streptomycin-glutamine,1× nonessential amino acid
Vero E6	-	American Type Culture Collection (VA, USA)	CRL1568	Split 1:20 twice a week	Eagle’s Minimum Essential Medium + 10% (*v/v*) Foetal Bovine Serum, 1× penicillin–streptomycin-glutamine,1× nonessential amino acids

## Data Availability

The original data presented in the study are openly available in figshare repository at https://doi.org/10.6084/m9.figshare.30618944.

## Supplementary Materials

The following supporting information can be downloaded at https://www.mdpi.com/article/10.3390/pharmaceutics17121584/s1: Figure S1: The characterisation of gene silencing in HEK293 (SC008) cells; Figure S2: The characterisation of HEK293 SC008 cells; Figure S3: The characterisation of subcellular fractionation in HEK293 and MCF-7 cells; Table S1: PCR primers used to amplify transgene contigs; Table S2: PCR products generated from HEK293 (SC008) genomic DNA; Table S3: Primers used to sequence PCR products; Table S4: Primers used in sequencing reactions.

## Abbreviations

The following abbreviations are used in this manuscript: antisense oligonucleotide (ASO), anthrax toxin (ATx), antisense oligonucleotides (ASO), diphtheria toxin a chain (DTA), early endosome (EE), emerald GFP (EmGFP), intraluminal vesicles (ILV), late endosome (LE), lethal factor (N-terminal) (LFn), LFn fused-in-frame with DTA (LFn-DTA), LFn fused-in-frame with PKR (LFn-PKR), green fluorescent protein (GFP), multivesicular body (MVB), phosphoinositide 3-kinases (PI3 kinase), protective antigen (PA) of 83 KDa (PA83), protective antigen of 63 KDa (PA63), plasma membrane (PM), post-nuclear supernatant (PNS), red fluorescent protein (RFP), RNA-binding domain of protein kinase R (PKR), small interfering (si) RNA, tandem dimeric tomato (TdTom), wheatgerm agglutinin (WGA).

## References

[1] Kerr J.R., Schneider C.R., Recchia G., Dryhurst S., Sahlin U., Dufouil C., Arwidson P., Freeman A.L., van der Linden S. (2021). Correlates of intended COVID-19 vaccine acceptance across time and countries: Results from a series of cross-sectional surveys. BMJ Open.

[2] Jo S.J., Chae S.U., Bin Lee C., Bae S.K. (2023). Clinical Pharmacokinetics of Approved RNA Therapeutics. Int. J. Mol. Sci..

[3] Lorenzer C., Dirin M., Winkler A.-M., Baumann V., Winkler J. (2015). Going beyond the liver: Progress and challenges of targeted delivery of siRNA therapeutics. J. Control. Release.

[4] Dyer P.D., Shepherd T.R., Gollings A.S., Shorter S.A., Gorringe-Pattrick M.A., Tang C.-K., Cattoz B.N., Baillie L., Griffiths P.C., Richardson S.C. (2015). Disarmed anthrax toxin delivers antisense oligonucleotides and siRNA with high efficiency and low toxicity. J. Control. Release.

[5] Hirschenberger M., Stadler N., Fellermann M., Sparrer K.M.J., Kirchhoff F., Barth H., Papatheodorou P. (2021). CRISPA: A Non-viral, Transient Cas9 Delivery System Based on Reengineered Anthrax Toxin. Front. Pharmacol..

[6] Verdurmen W.P.R., Luginbühl M., Honegger A., Plückthun A. (2015). Efficient cell-specific uptake of binding proteins into the cytoplasm through engineered modular transport systems. J. Control. Release.

[7] Liao X., Rabideau A.E., Pentelute B.L. (2014). Delivery of antibody mimics into mammalian cells via anthrax toxin protective antigen. ChemBioChem.

[8] Feron B.K.L., Gomez T., Youens N.C., Mahmoud N.A.M., Abdelrahman H.K.S., Bugert J.J., Richardson S.C.W. (2025). Antiviral siRNA Delivered Using Attenuated, Anthrax Toxin Protects Cells from the Cytopathic Effects of Zika virus. Virus Genes.

[9] Eden E.R., Huang F., Sorkin A., Futter C.E. (2011). The role of EGF receptor ubiquitination in regulating its intracellular traffic. Traffic.

[10] Abrami L., Lindsay M., Parton R.G., Leppla S.H., van der Goot F.G. (2004). Membrane insertion of anthrax protective antigen and cytoplasmic delivery of lethal factor occur at different stages of the endocytic pathway. J. Cell Biol..

[11] Jiang J., Pentelute B.L., Collier R.J., Zhou Z.H. (2015). Atomic Structure of Anthrax protective antigen pore elucidates toxin translocation. Nature.

[12] Milne J.C., Blanket S.R., Hanna P.C., Collier R.J. (1995). Protective antigen-binding domain of anthrax lethal factor mediates translocation of a heterologous protein fused to its amino- or carboxy-terminus. Mol. Microbiol..

[13] Muraki M., Ishimura M., Harata K. (2002). Interactions of wheat-germ agglutinin with GlcNAcβ, 6Gal sequence. Biochim. Biophys. Acta.

[14] Shorter S.A., Pettit M.W., Dyer P.D.R., Youngs E.C., Gorringe-Pattrick M.A.M., El-Daher S., Richardson S. (2017). Green Fluorescent Protein (GFP): Is seeing believing and is that enough?. J. Drug Target..

[15] Richardson S.C.W., Winistorfer S.C., Poupon V., Luzio J.P., Piper R.C. (2004). Mammalian Late Vacuole Protein Sorting Orthologues Participate in Early Endosomal Fusion and Interact with the Cytoskeleton. Mol. Biol. Cell..

[16] Patki V., Virbasius J., Lane W.S., Toh B.-H., Shpetner H.S., Corvera S. (1997). Identification of an early endosomal protein regulated by phosphatidylinositol 3-kinase (wortmannin). Proc. Natl. Acad. Sci. USA.

[17] Bright N.A., Reaves B.J., Mullock B.M., Luzio J.P. (1997). Dense core lysosomes can fuse with late endosomes and are re-formed from the resultant hybrid organelles. J. Cell Sci..

[18] Sava I., Davis L.J., Gray S.R., Bright N.A., Luzio J.P. (2024). Reversible assembly and disassembly of V-ATPase during the lysosome regeneration cycle. Mol. Biol. Cell.

[19] Babst M. (2011). MVB Vesicle Formation: ESCRT-Dependent, ESCRT-Independent and Everything in Between. Curr. Opin. Cell Biol..

[20] Schneider C., Rasband W., Eliceiri K. (2012). NIH Image to ImageJ: 25 years of image analysis. Nat. Methods.

[21] Richardson S.C.W., Wallom K.L., Ferguson E.L., Deacon S.P.E., Davies M.W., Powell A.J., Piper R.C., Duncan R. (2008). The use of fluorescence microscopy to define polymer localisation to the late endocytic compartments in cells that are targets for drug delivery. J. Control. Release.

[22] Richardson S.C.W., Pattrick N.G., Lavignac N., Ferruti P., Duncan R. (2010). Intracellular fate of bioresponsive poly(amidoamine)s in vitro and in vivo. J. Control. Release.

[23] Corrotte M., Fernandes M.C., Tam C., Andrews N.W. (2011). Toxin Pores Endocytosed During Plasma Membrane Repair Traffic into the Lumen of MVBs for Degradation. Traffic.

[24] Abrami L., Brandi L., Moayeri M., Brown M.J., Krantz B.A., Leppla S.H., van der Goot F.G. (2013). Hijacking multivesicular bodies enables long-term and exosome-mediated long-distance action of anthrax toxin. Cell Rep..

[25] Sergeeva O., van der Goot F. (2019). Converging physiological roles of the anthrax toxin receptors. F1000research.

[26] Abrami L., Leppla S.H., van der Goot F.G. (2006). Receptor palmitoylation and ubiquitination regulate anthrax toxin endocytosis. J. Cell Biol..

[27] Richardson S., Feron B. (2019). WO2020030923—Method for Preparing Liposomes.

[28] Arnold A.E., Smith L.J., Beilhartz G.L., Bahlmann L.C., Jameson E., Melnyk R.A., Shoichet M.S. (2020). Attenuated diphtheria toxin mediates siRNA delivery. Sci. Adv..

[29] Huotari J., Helenius A. (2011). Endosome maturation. EMBO J..

[30] Brown M.J., Thoren K.L., Krantz B.A. (2015). Role of the α Clamp in the Protein Translocation Mechanism of Anthrax Toxin. J. Mol. Biol..

[31] Moghimi S.M., Symonds P., Murray J.C., Hunter A., Debska G., Szewczyk A.C. (2005). A two-stage poly(ethylenimine)-mediated cytotoxicity: Implications for gene transfer/therapy. Mol. Ther..

[32] Richardson S.C.W., Kolbe H.V.J., Duncan R. (1999). Potential of low molecular mass chitosan as a DNA delivery system: Biocompatibility, body distribution and ability to complex and protect DNA. Int. J. Pharm..

[33] Sinden R.R., Pearson C.E., Potaman V.N., Ussery D.W. (1998). DNA: Structure and Function. Advances in Genome Biology.

[34] Beaufay H., de Duve C. (1954). The hexosephosphatase system. VI. Attempted fractionation of microsomes containing glucose-6-phosphatase. Bull. Soc. Chim. Biol..

[35] Zerial M., Melancon P., Schneider C., Garoff H. (1986). The transmembrane segment of the human transferrin receptor functions as a signal peptide. EMBO J..

[36] Gilleron J., Querbes W., Zeigerer A., Borodovsky A., Marsico G., Schubert U., Manygoats K., Seifert S., Andree C., Stöter M. (2013). Image-Based analysis of lipid nanoparticle–mediated sirNA delivery, intracellular trafficking and endosomal escape. Nat. Biotechnol..

[37] Dowdy S.F. (2017). Overcoming cellular barriers for RNA therapeutics. Nat. Biotechnol..

[38] Yang M., Jin H., Chen J., Ding L., Ng K.K., Lin Q., Lovell J.F., Zhang Z., Zheng G. (2011). Efficient Cytosolic Delivery of siRNA Using HDL-Mimicking Nanoparticles. Small.

[39] Hedlund H., Du Rietz H., Johansson J., Zedan W., Huang L., Wallin J., Wittrup A. (2021). Absolute quantification and single-cell dose-response of cytosolic siRNA delivery. Nat. Commun..

[40] Detzer A., Overhoff M., Wünsche W., Rompf M., Turner J.J., Ivanova G.D., Gait M.J., Sczakiel G. (2009). Increased RNAi is related to intracellular release of siRNA via a covalently attached signal peptide. RNA.

