# Supplementary material for: Intraluminal Vesicles as Transfection Intermediaries
Source: Pharmaceutics. 2025 Dec 9;17(12):1584. doi: 10.3390/pharmaceutics17121584 (PMC12737133; doi:10.3390/pharmaceutics17121584)
Supplement: Supplementary file 1 [file pharmaceutics-17-01584-s001.zip › pharmaceutics-3985251-supplementary.pdf]

pharmaceutics

MDPI

*Supplementary Materialse*

# Intraluminal Vesicles as Transfection Intermediaries

**Nourhan A. M. Mahmoud [1,3,†], Hadeer K. S. Abdelrahman [1,3,†], and Benedita K. L Feron [1, 4], Andra Pintilie [1] Marc Fivaz [4], Joanna J. Miest-Bray [2], Timothy Gomez [1], Natalie Youens [1], Vineeta Tripathi [5] and Simon C. W. Richardson [1*]**

[1] The Exogenix Laboratory, School of Science, University of Greenwich, Central Avenue, Chatham Maritime, Kent, ME4 4TB, UK.
[2] The School of Science, University of Greenwich, Central Avenue, Chatham Maritime, Kent, ME4 4TB, UK.
[3] Current address: Department of Biochemistry, Faculty of Pharmacy, October University for Modern Sciences and Arts (MSA), Giza 12451, Egypt.
[4] Current address: Umeå University, Sweden.
[4] Current address: Remynd, Bio-incubator, Gaston Geenslaan 1, 3001 Leuven, Belgium.
[5] Vitarka Therapeutics Ltd., Innovation House, Discovery Park Sandwich, Kent, CT13 9FF, United Kingdom.
† These Authors Contributed Equally to this work
* Correspondence: S.C.W.Richardson@Greenwich.ac.uk

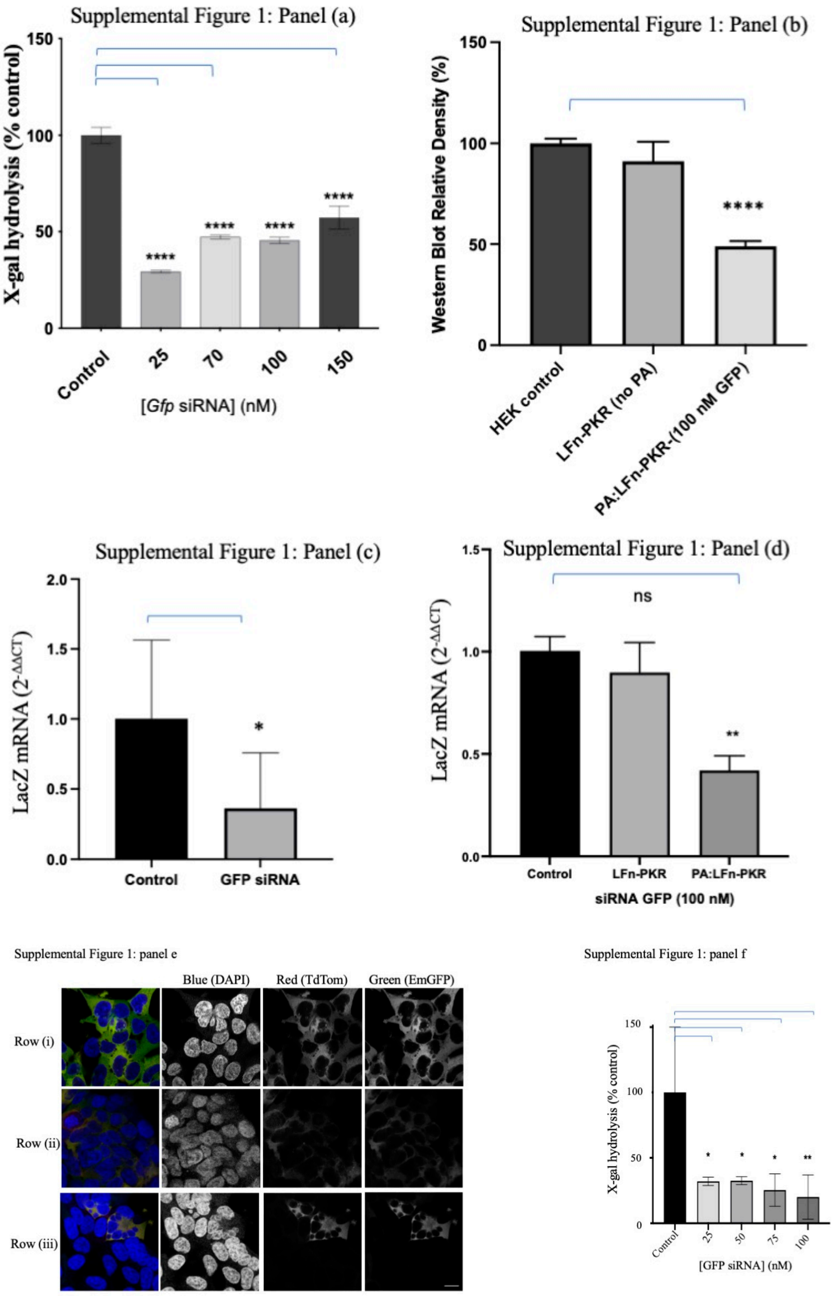


**Figure S1.** documents the characterisation of the HEK293$^{GFP-LacZ-RFP}$ (SC008) cells. Panel (a) X-gal hydrolysis 48h after Nucleofection® (n=3±SD). Here (**panel a**) target gene (GFP-LacZ) expression levels were measured by documenting X-gal hydrolysis 48h after Nucleofection®. These data were then reinforced by measuring gene (GFP-LacZ) expression relative to a housekeeper (GAPDH) using Western analysis 24h after transfection and are shown (**panel b**) (Transfection using PA and LFn-PKR Measured using Western Immunoblotting after 24h (representative of n=3±SD)) using HEK293 SC008 cells. Finally, LacZ-GFP mRNA transcript levels were assayed by using RTq-PCR after both Nucleofection and transfection with PA and LFn-PKR (**panel c** and **panel d**). Panel (c) Target mRNA levels in HEK293$^{Gfp\text{-}LacZ:Rfp}$ cells 24h after Nucleofection® with *Gfp* siRNA (n=9±SD) and Panel (c) Target mRNA levels in HEK293$^{Gfp\text{-}LacZ:Rfp}$ cells 24h after Nucleofection® with *Gfp* siRNA (n=9±SD). .**Panel (e)** documents the characterisation of transgene expression in HEK293$^{Gfp\text{-}LacZ:Rfp}$ cells by confocal fluorescence microscopy. Row (i) Scrambled *Gfp* siRNA delivered by PA83::LFn-PKRROw (ii) *Gfp* targeting siRNA delivered by Nucleofection®. RPw (iii) (iii) *Gfp* targeting siRNA delivered by PA83::LFn-PKR. **Panel (f)** X-gal hydrolysis 48h after PA::LFn-PKR :siRNA transfection (n=3±SD). **Statistics**. X-Gal hydrolysis assay (**panel a**) Nucleofection®

vs:25nM siRNA $p<0.0001$ ****, 70 nM siRNA $p<0.0001$ ****, 100nm siRNA<0.0001 ****, 150nM siRNA $p<0.0001$ ****. **Panel b**: Western Immunoblotting, 100nM siRNAp<0.00001 ****, By Nucleofection® and RTq-PCR, p-0.015 *, by PA:LFn-PKR and RTqPCR $p=0.001$ **. For characterising X-gal hydrolysis at 48h (**panel f**), control 90nM siRNA vs 25nM siRNA $p=0.0213$ *, 70nM siRNA $p=0.0221$ *, 100nM siRNA $p=0.0124$ *, 150nM siRNA $p=0.0081$ **.

(panel a): Contiguous assembly of sequenced PCR products

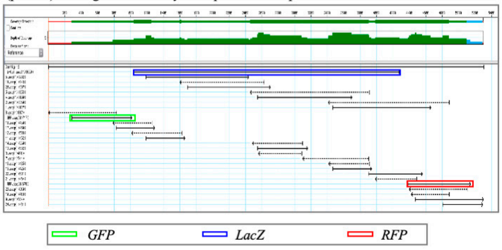


To this end, genomic DNA was prepared from 2 x 75 $cm^2$ flasks of HEK293$^{Gfp\text{-}LacZ:Rfp}$ (SC008) cells at 95% confluence us ing a DNeasy Blood & Tissue Kit (QIAGEN, Manchester, UK). This genomic DNA was used as a template and the tran sgene amplified using DreamTaq (ThermoFisher Scientific, Paisley, UK), following the manufacturer's instructions. The primer pairs listed (Table S1) were used to produce the PCR products detailed (Table S2, Figure S2 (panel a)). Sequencing required the removal of excess dNTPs from the PCR products which was performed using a QIAquick PCR Purification kit (QIAGEN, Manchester, UK). Sequencing reactions were then performed using the primers listed (Table S3) and the PCR products previously documented (Table S2). Sequencing was performed at the University of Dundee MRC PPU DNA sequencing and services unit (https://www.dnaseq.co.uk). Sequencing reactions (Table S4) were assembled using SeqManPro, part of the DNAStar LaserGene software suite (Madison, WI, USA).

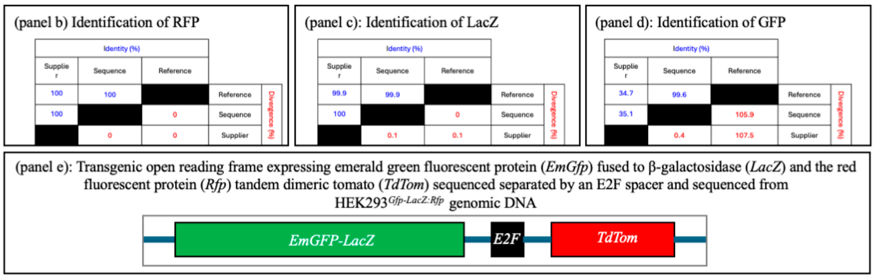


After mapping the open reading frame and translating the encoded protein *in silico*, Megalign Pro software (LaserGene), using Clustal-W algorithm, was used to search for the specific identity of the encoded proteins against all the known translated LacZ, RFP and GFP variants (available at www.snapgene.com). The annotated assembled sequence was submitted to and is accessible through Genbank (MH015340.1) (Figure S1 (panel e)). To corroborate these data, live cell imaging was undertaken using the filter sets identified as being optimal for EmGFP and TdTom (RFP) (Figure S2). Western blotting was also performed and the GFP-LacZ and TdTom proteins identified using RFP and GFP specific antibodies (Table 3) at the predicted molecular weight (data not shown).

Before developing an assay to rapidly assay gene silencing in the stably transfected HEK293$^{Gfp\text{-}LacZ:Rfp}$ (SC008) cells, it was necessary to sequence the transgene to ensure the specificity of the siRNA used to mediate knockdown, to ensure the nature of the fluorescent proteins in order to use the optimal laser lines when examining them by confocal fluorescence microscopy and to ensure the correct antibodies and probes were being used for western analysis and RT-qPCR.

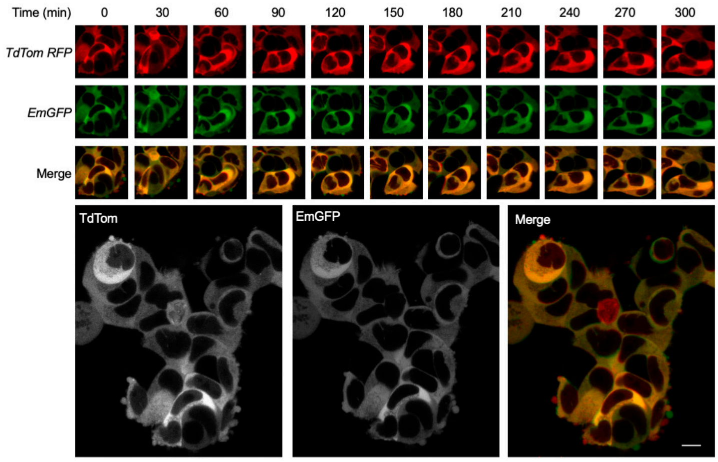


(panel e). Live Cell Imaging of HEK293$^{Gfp\text{-}LacZ:Rfp}$ growing *in vitro:* https://youtu.be/-EQfRHBNpkw

(b

**Figure S2.** panel **a**: Sequencing and assembling the transgene in SC008 cell. panel (**b**). Identifying the transgene components. panel **e**, Live cell imaging of SC008 cells

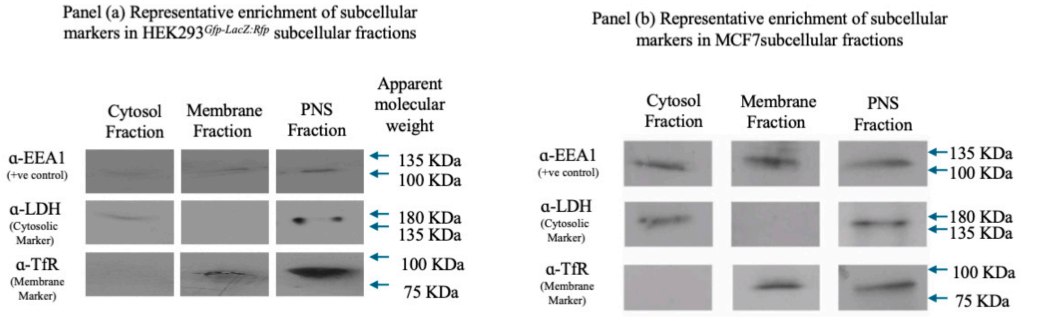


Figure (S3) shows the representative characterisation of marker distribution using Western analysis in both HEK293$^{Gfp\text{-}LacZ:Rfp}$ (SC008) (panel a) or MCF7 cells (panel b) after subcellular fractionation. Here the transmembrane protein (transferrin receptor (TfR) was seen in both instances to be detected exclusively in the PNS and membrane fractions. Conversely the cytosol marker Lactate dehydrogenase was found exclusively in the cytosol fraction denoting appropriate separation and enrichment of the two compartments.

**Figure S3.** Characterization of subcellular fractions.

**Table S1.** PCR primers used to amplify HEK293 (SC008) genomic DNA

| Primer name | Oligonucleotide sequence (5′) |
|---|---|
| GFP-LacZ F | ttc aag acc cgc cac aac at |
| GFP-LacZ R | acg acg aca gta tcg gcc tca g |
| LacZ Mid F | tgt ggc gga tga gcg gca |
| LacZ Mid R | tcc acg cgc gcg tac atc |
| LacZ RFP F | tgg cct gaa ctg cca gct |
| LacZ RFP R | cag ctt ggc ggt ctg ggt |
| End RFP F | agc ctg cag gac ggc agc t |
| End RFP R | aca ggt ggt gcc ggc cct |
| GFP F002 | tgg tga gca agg gcg agg ag |
| GFP R694 | gta cag ctc gtc cat gcc gag |
| RFP F004 | gcc agc agc gag gac gtg atc |
| RFP R481 | ctt cag ctt cag ggc ctt gtg gat |
| LacZ F117 | cac cga tcg ccc ttc cca aca gt |
| LacZ R592 | tgc cgc tca tcc gcc aca t |
| LacZ F1370 | at cag gcc acg gcg cta atc acg |
| LacZ F2256 | gca att taa ccg cca gtc agg |
| LacZ R1946 | tac cat cca gcg cca cca tcc agt |
| LacZ R2695 | agt aag gcg gtc ggg ata gtt ttc ttg |
| GFP-LacZ F | ttc aag acc cgc cac aac at |

**Table S2.** PCR products generated from HEK293$^{Gfp\text{-}LacZ:Rfp}$ genomic DNA.

| PCR product | 5′ Primer | 3′ Primer | Size (bp) |
|---|---|---|---|
| LacZ-RFP | LacZ F2256 | RFP R481 | 1264 |
| GFP | GFP F002 | GFP R694 | 692 |
| RFP | RFP F004 | RFP R481 | 477 |

| | | | |
|---|---|---|---|
| LacZ 1 | LacZ F117 | LacZ R592 | 494 |
| LacZ 2 | LacZ F1370 | LacZ R1946 | 576 |
| LacZ 3 | LacZ F2256 | LacZ R2695 | 462 |
| LacZ 4 | LacZ F117 | LacZ R1946 | 1829 |
| LacZ 5 | LacZ F1370 | LacZ R2695 | 1325 |
| LacZ 6 | LacZ F117 | LacZ R2695 | 2578 |
| GFP-LacZ | GFP-LacZ F | GFP-LacZ R | 467 |
| LacZ Mid | LacZ Mid F | LacZ Mid R | 916 |
| LacZ RFP | LacZ RFP F | LacZ RFP R | 565 |
| End RFP | End RFP F | End RFP R | 337 |
| LacZ-RFP | LacZ F2256 | RFP R481 | 1264 |

**Table S3.** Primers used to sequence PCR products.

| Primer | Oligonucleotide Sequence |
|---|---|
| GFP-LacZ F | ttc aag acc cgc cac aac at |
| GFP-LacZ R | acg acg aca gta tcg gcc tca g |
| LacZ Mid F | tgt ggc gga tga gcg gca |
| LacZ Mid R | tcc acg cgc gcg tac atc |
| LacZ RFP F | tgg cct gaa ctg cca gct |
| LacZ RFP R | cag ctt ggc ggt ctg ggt |
| End RFP F | agc ctg cag gac ggc agc t |
| End RFP R | aca ggt ggt gcc ggc cct |
| GFP F002 | tgg tga gca agg gcg agg ag |
| GFP R694 | gta cag ctc gtc cat gcc gag |
| RFP F004 | gcc agc agc gag gac gtg atc |
| RFP R481 | ctt cag ctt cag ggc ctt gtg gat |
| LacZ F117 | cac cga tcg ccc ttc cca aca gt |
| LacZ R592 | tgc cgc tca tcc gcc aca t |
| LacZ F1370 | at cag gcc acg gcg cta atc acg |
| LacZ F2256 | gca att taa ccg cca gtc agg |
| LacZ R1946 | tac cat cca gcg cca cca tcc agt |
| LacZ R2695 | agt aag gcg gtc ggg ata gtt ttc ttg |

**Table S4.** Sequencing reactions.

| Sequencing Primer | PCR Product Template | Designation (Supplemental Figure 2: Panel a) |
|---|---|---|
| LacZ F2256 | LacZ-RFP | 1 |
| RFP R481 | LacZ-RFP | 2 |
| LacZ R1946 | LacZ 4 | 3 |
| LacZ F1370 | LacZ 5 | 4 |
| LacZ R2695 | LacZ 5 | 5 |
| LacZ F117 | LacZ 6 | 6 |
| LacZ R2695 | LacZ 6 | 7 |
| GFP R556 | GFP | 8 |
| RFP F034 | RFP | 9 |
| RFP R481 | RFP | 10 |
| LacZ F117 | LacZ 1 | 11 |
| LacZ R592 | LacZ 1 | 12 |
| LacZ F1370 | LacZ 2 | 13 |
| LacZ R1946 | LacZ 2 | 14 |

| | | |
|---|---|---|
| LacZ F2256 | LacZ 3 | 15 |
| LacZ R2695 | LacZ 3 | 16 |
| GFP-LacZ F | GFP-LacZ | 17 |
| GFP-LacZ R | GFP-LacZ | 18 |
| LacZ Mid F | LacZ Mid | 19 |
| LacZ Mid R | LacZ Mid | 20 |
| LacZ RFP F | LacZ-RFP | 21 |
| LacZ RFP R | LacZ-RFP | 22 |
| End RFP F | End RFP | 23 |
| End RFP R | End RFP | 24 |
